# Dietary Gluten-Induced Gut Dysbiosis Is Accompanied by Selective Upregulation of microRNAs with Intestinal Tight Junction and Bacteria-Binding Motifs in Rhesus Macaque Model of Celiac Disease

**DOI:** 10.3390/nu8110684

**Published:** 2016-10-28

**Authors:** Mahesh Mohan, Cheryl-Emiliane T. Chow, Caitlin N. Ryan, Luisa S. Chan, Jason Dufour, Pyone P. Aye, James Blanchard, Charles P. Moehs, Karol Sestak

**Affiliations:** 1 Division of Comparative Pathology, Tulane National Primate Research Center, Covington, LA 70433, USA; mmohan@tulane.edu (M.M.); paye@tulane.edu (P.P.A.); 2 Second Genome Inc., San Francisco, CA 94080, USA; cheryl@secondgenome.com (C.-E.T.C.); caitlin@secondgenome.com (C.N.R.); luisa@secondgenome.com (L.S.C.); 3 Division of Veterinary Resources, Tulane National Primate Research Center, Covington, LA 70433, USA; jdufour@tulane.edu (J.D.); jblanch1@tulane.edu (J.B.); 4 Arcadia Biosciences Inc., Seattle, WA 98119, USA; max.moehs@arcadiabio.com; 5 Division of Microbiology, Tulane National Primate Research Center, Covington, LA 70433, USA; 6 PreCliniTria LLC, Mandeville, LA 70471, USA

**Keywords:** celiac, gluten, gut, microbiome, microbiota, dysbiosis, rhesus, macaque, metagenomics, 16S rRNA, miRNA, chronic inflammation

## Abstract

The composition of the gut microbiome reflects the overall health status of the host. In this study, stool samples representing the gut microbiomes from 6 gluten-sensitive (GS) captive juvenile rhesus macaques were compared with those from 6 healthy, age- and diet-matched peers. A total of 48 samples representing both groups were studied using V4 16S rRNA gene DNA analysis. Samples from GS macaques were further characterized based on type of diet administered: conventional monkey chow, i.e., wheat gluten-containing diet (GD), gluten-free diet (GFD), barley gluten-derived diet (BOMI) and reduced gluten barley-derived diet (RGB). It was hypothesized that the GD diet would lower the gut microbial diversity in GS macaques. This is the first report illustrating the reduction of gut microbial alpha-diversity (*p* < 0.05) following the consumption of dietary gluten in GS macaques. Selected bacterial families (e.g., *Streptococcaceae* and *Lactobacillaceae*) were enriched in GS macaques while *Coriobacteriaceae* was enriched in healthy animals. Within several weeks after the replacement of the GD by the GFD diet, the composition (beta-diversity) of gut microbiome in GS macaques started to change (*p* = 0.011) towards that of a normal macaque. Significance for alpha-diversity however, was not reached by the day 70 when the feeding experiment ended. Several inflammation-associated microRNAs (miR-203, -204, -23a, -23b and -29b) were upregulated (*p* < 0.05) in jejunum of 4 biopsied GS macaques fed GD with predicted binding sites on 16S ribosomal RNA of *Lactobacillus reuteri* (accession number: NR_025911), *Prevotella stercorea* (NR_041364) and *Streptococcus luteciae* (AJ297218) that were overrepresented in feces. Additionally, claudin-1, a validated tight junction protein target of miR-29b was significantly downregulated in jejunal epithelium of GS macaques. Taken together, we predict that with the introduction of effective treatments in future studies the diversity of gut microbiomes in GS macaques will approach those of healthy individuals. Further studies are needed to elucidate the regulatory pathways of inflammatory miRNAs in intestinal mucosa of GS macaques and to correlate their expression with gut dysbiosis.

## 1. Introduction

The human gastrointestinal (GI) tract contains approximately 10^14^ microorganisms [1] that colonize a surface of >30 m^2^ [2]. The gut microbiome co-exists with its host as a super-organism, in a mutualistic manner [3,4,5], affecting the host’s metabolism, immunity and overall fitness [6,7]. Diet, age, gender, genetics, usage of antibiotics, and multiple other factors influence the composition of the gut microbiome [8,9,10,11,12,13,14,15,16,17]. 

Non-human primates, owing to their close biological similarity with humans, are a valuable resource in biomedical research [18]. An earlier study by McKenna and colleagues identified important similarities but at the same time unique differences between human and rhesus gut microbiomes [19]. For example, *Treponema* sp. spirochetes were found to be abundant in macaques [19]. Recent studies with rural African human populations revealed an overabundance of intestinal *Treponoma* and *Prevotella* sp. compared to populations consuming a Western type of diet [16,20,21]. McKenna and colleagues documented an alteration in the composition of the gut microbiome, i.e., intestinal dysbiosis in rhesus monkeys, due to chronic colitis [19]. In another study, utilizing infant macaques that were either breast- or bottle-fed, differences in immune responsiveness and accumulation of metabolites were noted and linked to changes in gut microbiome composition [22]. In Japanese macaques (*Macaca fuscata*), consumption of a high-fat maternal diet resulted in displacement of potentially harmful gut microflora such as *Campylobacter* sp. [15]. Finally, inulin treatment successfully resolved idiopathic chronic diarrhea and restored gut microflora in dysbiotic macaques [23,24]. 

A loss of gut microbial diversity as one of the hallmarks of dysbiosis is commonly found in patients with Inflammatory Bowel Disease (IBD). While many obligate anaerobic commensal microorganisms are lost during IBD, an increase of aerotolerant *Enterobacteriaceae* and expansion of the *Prevotellaceae* takes place [25,26,27,28,29,30]. Intestinal dysbiosis has also been observed in patients with celiac disease (CD) [31,32,33,34,35]. Investigations that focused on pediatric patients during and after the Swedish CD outbreak, suggested that rod-shaped intestinal bacteria might have predisposed children to CD [36,37,38]. It has been reported that bacteria most involved in gluten metabolism belong to phylum *Firmicutes*, mainly from the *Lactobacillus* genus, followed by *Streptococcus*, *Staphylococcus* and *Clostridium* genera [39]. It was shown that GFD treatment significantly alters proportions of these bacterial populations [31]. It was suggested that increased presence of some of the bacteria involved in gluten metabolism might be associated with enteritis [39]. An unrelated report showed that *Proteobacteria* and not *Firmicutes* is the most abundant phylum in celiacs, with members of the *Neisseria* genus being the most represented [40]. Regardless of the exact reflection of intestinal dysbiosis that appears may vary in different categories of CD patients, it was observed that dietary gluten-induced dysbiosis is not easily restored by GFD treatment [35]. Although in GS rhesus macaques the progression of enteropathy is linked with the gradually decreasing presence of mucosal barrier-maintaining interleukins (IL)-17, IL-22 [41] and various other functions, alterations in gut microbiota are yet to be studied in this model. Since a recent study demonstrated that fecal miRNAs secreted by intestinal epithelial cells could enter luminal bacterial cells and regulate their growth via post-transcriptional gene regulation [42], we profiled miRNA expression in jejunum of GS macaques. Thirteen differentially expressed (DE) miRNAs were identified, with eight containing specific binding motifs to dysbiotic bacterial species and intestinal tight junction (TJ) proteins. In summary, our main objective was to determine if dysbiosis takes place in GS macaques fed a gluten-containing diet and if it can be restored upon administration of GFD. Results indicate that the diversity of the gut microbiome of GS macaques is significantly lower than that of healthy, age-matched peers and dysbiosis is linked with upregulation of pro-inflammatory miRNAs. Future studies shall focus on restoration of gut microbiome diversity and composition—by a long-term dietary and/or other therapeutic interventions. 

## 2. Experimental Section

### 2.1. Ethics Approval

This study was performed using samples collected from normal healthy and GS non-human primates. Ethics approval for veterinary procedures was obtained from the Tulane University Animal Care and Use Committee, Animal Welfare Assurance A-4499-01. Tulane National Primate Research Center (TNPRC) is accredited by the Association for Assessment and Accreditation of Laboratory Animal Care (AAALAC). Steps were taken to ameliorate animal suffering in accordance with the recommendations of the Guide to the Care and Use of Laboratory Animals (NIH) 78-23 (Revised, 2011). 

### 2.2. Rhesus Macaques, Diets and Samples Collected 

Forty-eight stool samples were obtained via fecal loops from 12 juvenile (1–3-years-old, 6 healthy controls and 6 GS) captive rhesus macaques (*Macaca mulatta*) of Indian origin. As described, the GS and control macaques were stationed in a dedicated bio-security level 2 facility, physically separated from the rest of the colony as well as from the same study animals on different, dietary gluten-modified diets, to prevent contamination of each chow with undesirable gluten sources [43]. The 12 animals were selected irrespective of sex. All animals were seronegative and free of viral, bacterial or parasitic pathogens including the simian retrovirus type D, simian T lymphotropic virus type 1, simian immunodeficiency virus and herpes B virus [41,44]. Tuberculin skin tests were negative for each individual. The 6 GS macaques had previously been reported with celiac-like GS, i.e., an equivalent of human CD [45]. Approximately 0.5 g of stool were obtained from at least 3 representative macaques at each time point when fed gluten-modified diets: conventional monkey chow, i.e., wheat gluten-containing diet (GD), gluten-free diet (GFD), conventional barley gluten-derived diet (BOMI) and reduced gluten barley-derived diet (RGB) (Supplementary Materials Table S1). Samples from GS macaques were obtained at multiple time points while samples from healthy control macaques were obtained only once. Immediately upon collection, stools were suspended in 1.0 mL of phosphate saline buffer and then stored at −80 °C until processed for DNA extraction. 

### 2.3. DNA Extraction, Library Preparation and Profiling

Frozen stool samples were thawed at room temperature prior to DNA extraction. Approximately 0.25 g (wet weight) of stool was measured for each sample and DNA was extracted using the MoBio PowerMag Microbiome kit (Mo Bio Laboratories Inc., Carlsbad, CA, USA) according to the manufacturer’s guidelines and optimized for high-throughput processing. All samples were quantified using the Qubit Quant-iT dsDNA High Sensitivity Kit (Invitrogen, Life Technologies, Grand Island, NB, USA). To enrich samples for bacterial 16S V4 rDNA region, DNA was amplified utilizing fusion primers designed against the surrounding conserved regions tailed with sequences to incorporate Illumina (San Diego, CA, USA) adapters and indexing barcodes [46]. Each sample was PCR amplified with two differently bar coded V4 fusion primers. Samples that met the post-PCR quantification minimum were advanced for pooling and sequencing. For each sample, amplified products were concentrated using a solid-phase reversible immobilization method for the purification of PCR products and quantified by qPCR. An amplicon pool containing 16S V4 enriched, amplified, barcoded samples, was sequenced for 2 × 250 cycles on an Illumina MiSeq (San Diego, CA, USA). Samples were processed in a Good Laboratory Practices (GLP) compliant service laboratory running Quality Management Systems for sample and data tracking. 

### 2.4. Operational Taxonomic Unit (OTU) Selection

Sequenced paired-end reads were merged using USEARCH and the resulting sequences were compared as described [47,48]. Briefly, all sequences matching a unique strain with an identity ≥99% were assigned a strain OTU. To ensure specificity of the strain hits, a difference of ≥0.25% between the identity of the best hit and the second hit was required (e.g., 99.75 vs. 99.5). For each strain OTU, one of the matching reads was selected as a representative and all sequences were mapped by USEARCH (usearch_global) against the strain OTU representative sequence to calculate strain abundance. The remaining non-strain sequences were quality filtered and de-replicated with USEARCH. Resulting unique sequences were then clustered at 97% by UPARSE de novo OTU clustering and a representative consensus sequence per de novo OTU was determined. The UPARSE clustering algorithm includes a chimera filtering step. Representative OTU sequences were classified via mothur’s Bayesian classifier with a threshold of 80% confidence; the classifier was trained against the Greengenes reference database (v13.5, greengenes.lbl.gov) [49] of 16S rRNA sequences clustered at 99% similarity. Spurious OTUs were removed. 

### 2.5. Alpha- (within Sample) and Beta- (between Samples) Diversity

“Observed” diversity reflects the number of unique OTUs within each sample while Shannon diversity reflects the richness of a sample along with the relative abundance of present OTUs. Both Observed and Shannon diversities were used to assess alpha-diversity. The Bray-Curtis dissimilarity index was evaluated to determine beta-diversity. 

### 2.6. Ordination and Clustering 

Dendrograms were constructed to graphically summarize the inter-sample relationships based on Bray-Curtis dissimilarity using hierarchical clustering by Ward’s method. 

### 2.7. Whole Microbiome and Taxon Significance Testing

Permutational Analysis of Variance (PERMANOVA) was utilized for whole microbiome beta-diversity differences among discrete categorical or continuous variables [50]. Univariate differential abundance of OTUs was tested using a negative binomial noise model for the overdispersion and Poisson process intrinsic to this data, as implemented in the DESeq2 package [51], and described for microbiome applications [52].

### 2.8. miRNA Profiling, Real Time qRT-PCR and Confocal Microscopy

Proximal jejunum biopsy tissues from 4 GS and 6 healthy control macaques fed GD for at least one year (long-term GD) were collected and processed as described [45]. Half of the collected biopsies were preserved in 5 mL of RNA-later solution (Qiagen Inc., Valencia, CA, USA) while second half was embedded in paraffin and 7 μm sections were used for immunofluorescent staining, i.e., confocal microscopy. 

Total RNA from intact jejunal tissue samples was isolated using the miRNeasy total RNA isolation kit (Qiagen Inc.) following the manufacturer’s protocol. The 100 ng of total RNA was first reverse transcribed using the miRNA reverse transcription reaction kit and loaded onto the TaqMan^®^ OpenArray^®^ Human MicroRNA Panel, QuantStudio™ 12K Flex system (Thermo-Fisher, Waltham, MA, USA) and processed as described previously [53]. 

TJ protein (Claudin-1, Claudin-3 and Occludin gene expression in jejunum samples was quantified by Power SYBR Green RNA to C_T_ One-Step RT-PCR assay (Thermo-Fisher). Each qRT-PCR reaction (20 μL) contained the following: 2X Power SYBR Green Master Mix (12.5 μL), 200 nM forward and reverse primer (Supplementary Materials Table S2) and 200 ng of total RNA. Comparative real-time PCR was performed and relative change in gene expression was calculated using the ΔΔCT method. Data was normalized to a combination of three endogenous controls (Beta-Actin, 18S rRNA and GAPDH). 

Immunofluorescence studies for the detection of Claudin-1 (1 in 50) (Abcam, Cambridge, UK) was done as described earlier [53]. Cytokeratin (Biocare, Concord, CA, USA) (1 in 500) and Topro-3 (1 in 2000) was employed as a marker for intestinal epithelial cells and nuclei, respectively. Positive signals were detected using appropriate Alexa fluor conjugated secondary antibodies (Thermo-Fisher, Waltham, MA, USA). 

### 2.9. Data Analysis

QuantStudio™ run files from GS (*n* = 4) and control macaques (*n* = 5) were analyzed simultaneously using ExpressionSuite software v1.0.3 (Thermo-Fisher) as described previously [54]. Since Expression Suite software is not equipped to perform non-parametric analysis, the output file containing five columns (well, sample, detector, task and C_T_ values) were saved as a tab-delimited text file, imported and analyzed by non-parametric Wilcoxon’s rank sum test for independent samples using RealTime STATMINER™ package (Integromics on Spotfire DecisionSite) designed to compare samples using the ∆∆C_T_ method for relative quantification of gene expression. miRNA expression data was normalized to a combination of two endogenous controls (RNU44 and RNU48). In all experiments, the C_T_ upper limit was set to 28 meaning that all miRNA detectors with a C_T_ value greater than or equal to 28 were excluded. TaqMan OpenArray^®^ microRNA data files were deposited with the National Center for Biotechnology Information database (GEO, Accession number: GSE89170, http://www.ncbi.nlm.nih.gov/geo/query/acc.cgi?acc=GSE89170).

For TJ protein mRNA qRT-PCR studies, one GS macaque with the highest ∆C_T_ value served as the calibrator/reference and assigned a value of 1. All DE mRNAs in GS and other macaques in the normal healthy control group are shown as an n-fold difference relative to this macaque. mRNA qRT-PCR data was analyzed by non-parametric Wilcoxon’s rank sum test for independent samples using RealTime STATMINER™ package. A *p* value of less than 0.05 was considered significant.

## 3. Results

### 3.1. Gut Microbiomes Differ Significantly between Healthy and GS Macaques 

In order to compare gut microbiomes between healthy and GS macaques that were fed conventional, gluten-containing monkey chow (GD) for at least one year, we measured alpha-diversity, relative abundance of top bacterial families and performed clustering analyses. 

**Figure 1:**
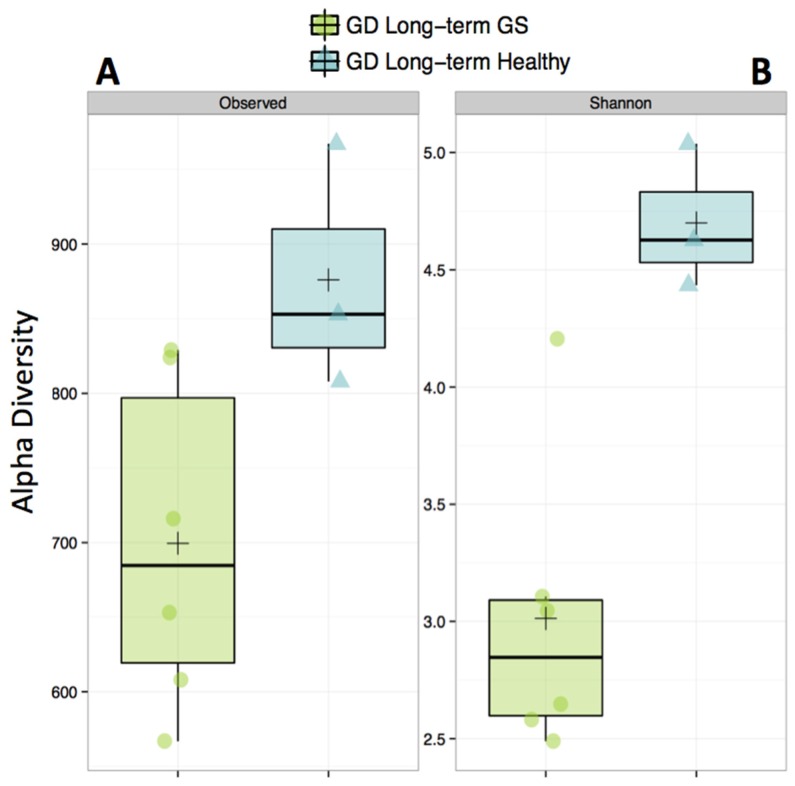
Alpha-diversity of the gut microbiome is decreased in gluten-sensitive (GS) macaques. Observed (**A**) corresponds to total number of Operational Taxonomic Units (OTUs) present in sample; Shannon, (**B**) corresponds to Shannon Diversity Index that accounts for both the abundance and evenness of OTUs. **Green** color represents GS juvenile macaques on GD (long-term) diet while **blue** indicates age- and gluten-containing diet (GD) diet-matched healthy controls.

**Figure 2:**
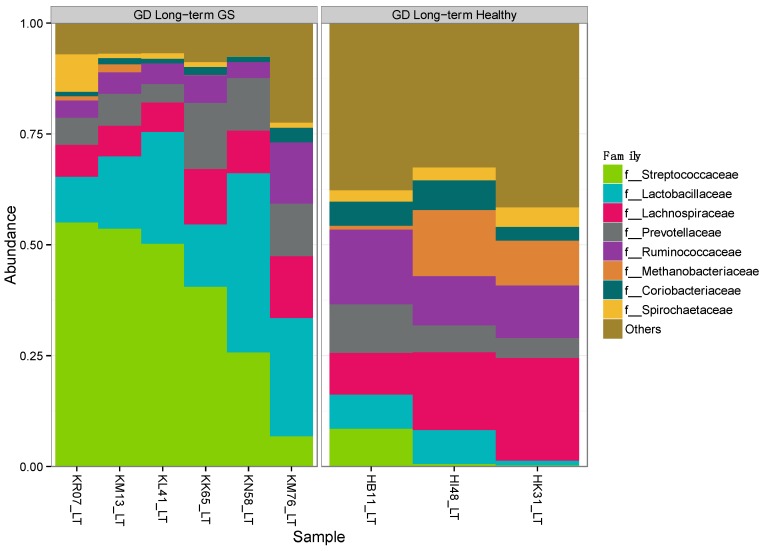
Proportional abundance of microbial taxa in GS and control macaques. Plot shows the most abundant taxa at the family level. **Left** panel represents GS juvenile macaques on GD diet while **right** panel indicates age- and diet-matched healthy control macaques.

Alpha-diversity (Shannon diversity index) was significantly higher in healthy compared to GS macaques (*p* = 0.02), despite that the observed number of OTUs did not differ (*p* = 0.07) (Figure 1, Supplementary Materials Table S3). Proportionally, two of the top 8 families (*Streptococcaceae* and *Lactobacillaceae*) were enriched in GS macaques, while one family (*Coriobacteriaceae*) was enriched in healthy macaques (Figure 2, Supplementary Materials Table S4). When gut microbial diversity metrics were compared between the GS and healthy animals with consideration of sex, there were similar differences as there were without such consideration.

**Figure 3:**
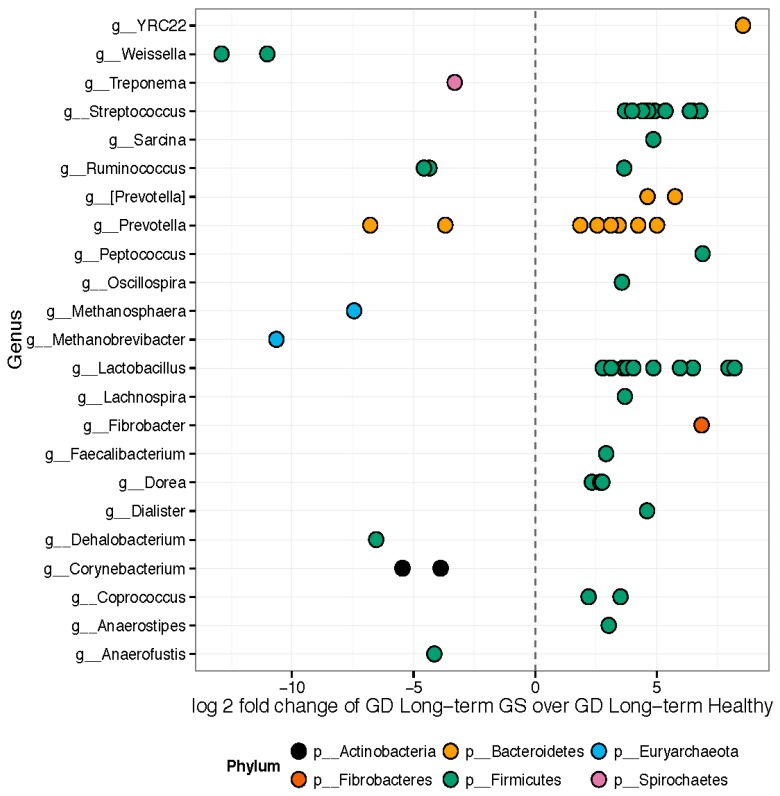
Differentially abundant features: GS macaques vs. healthy controls on GD. Each point represents an OTU belonging to each Genus. Only significant OTUs assigned at genus-level are shown. Features were considered significant if their FDR-corrected *p*-value was less than or equal to 0.05. There were 157 significantly different OTUs detected out of 1263 tested. Fifty-six OTUs had genus-level annotations.

Hierarchical clustering (Supplementary Materials Figure S1) and weighted ordination analyses showed good separation of represented bacterial families between samples collected from healthy control and GS macaques. The 157 OTUs differed significantly in relative abundance between healthy and GS macaques (Figure 3). Approximately 89 out of the 157 significant OTUs belonged to the phylum *Firmicutes*. Genera enriched in GS animals included *Anaerostipes*, *Coprococcus*, *Dorea*, *Feacalibacterium*, *Fibrobacter*, *Lachnospira*, *Lactobacillus*, *Oscillospira*, *Peptococcus*, *Prevotella*, *Ruminococcus*, *Sarcina*, *Streptococcus*, and YRC22. Genera enriched in healthy rhesus macaques included *Anaerofustis*, *Corynebacterium*, *Dehalobacterium*, *Methanobrevibacter*, *Methanosphaera*, *Prevotella*, *Ruminococcus*, *Treponema*, and *Weissella*. 

### 3.2. Gut Microbiomes of GS Macaques Are Influenced by GFD 

**Figure 4:**
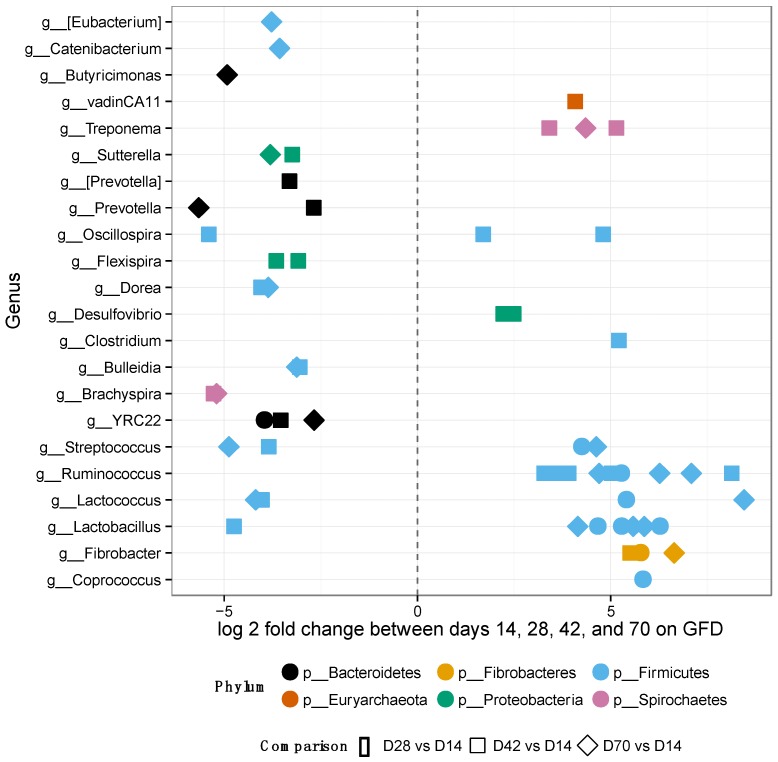
Differentially abundant features in GS macaques while on GFD. One hundred forty-five significantly different OTUs belonging to each genus out of 1212 tested were identified in GS macaques while on GFD. Overall increase of beneficial bacterial groups is seen in GS macaques while on GFD. Most of the significant features were from the phylum *Firmicutes*, particularly the family *Ruminococcaceae*.

After placing the GS macaques on GFD, alpha-diversity and relative OTU abundances were evaluated at days 14, 28, 42 and 70 to assess the extent of potential improvement, i.e., restoration of gut microbiome composition to that observed in normal healthy controls (Figure 4 and Supplementary Materials Figure S2). No significant differences were observed in alpha-diversity metrics by day 70 of GFD (Supplementary Materials Figure S2A,B).

**Figure 5:**
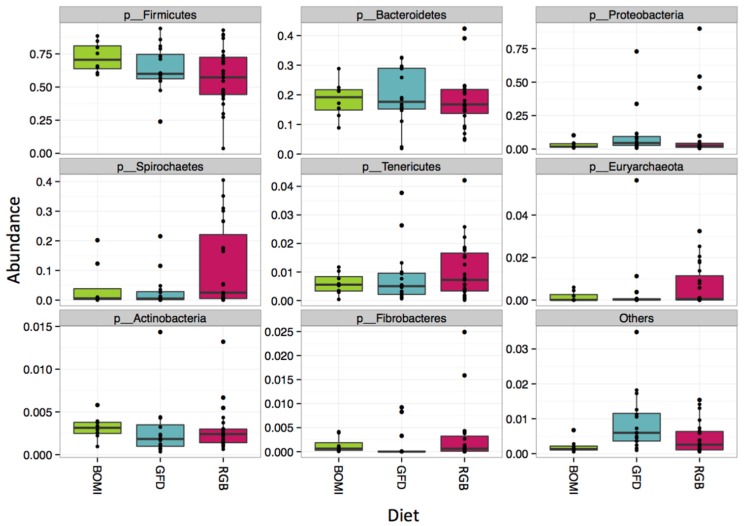
Composition at the phylum level. Irrespective of diet, the phylum *Firmicutes* comprised the majority of the gut microbiome in GS macaques.

Nevertheless, 145 of 1212 OTUs were significantly different in their abundance when the individual GFD time-points were tested (Figure 4). Many of the significant OTUs (23) belonged to families *Ruminococcaceae* and *Lachnospiraceae* (8) within the phylum *Firmicutes* (Figure 4, Supplementary Materials Table S5). In addition, there were no significant differences in alpha-diversity between BOMI and RGB diets (Supplementary Materials Figure S2C,D). Irrespective of the diet fed to GS macaques, the phylum *Firmicutes* comprised majority of the microbial species (mean = 64.7%). The phylum *Proteobacteria* was in a few instances dominant (mean = 7.8%) over *Firmicutes*. The relative abundances of the top 8 most abundant phyla did not differ significantly when comparing the GFD, BOMI and RGB diets. However, *Firmicutes*, followed by *Bacteroidetes* and *Proteobacteria* were dominant (Figure 5). 

While on GFD, significant changes in abundance of individual OTUs were detected in GS macaques (Figure 4): 145 differentially abundant OTUs of 1212 tested were identified. Overall, increase of beneficial bacterial groups was also seen in GS macaques while on GFD. Most of the significant OTUs were from the phylum *Firmicutes*, particularly the family *Ruminococcaceae*. The greatest number of significantly differential OTUs occurred between day 14 and 42 of GFD. Only 17 OTUs differed between day 14 and 28. Eleven OTUs were classified to a strain-level as *Brachyspira pilosicoli*, *Clostridium bartletti*, *Clostridium perfringens*, *Coprococcus eutactus*, *Desulfovibrio piger*, *Eubacterium biforme*, *Eubacterium siraeum*, *Flexispira/Helicobacter fennelliae*, *Oscillospira/Ruminococcaceae* bacterium D16, *Ruminococcus champanellensis*, and *Treponema berlinense*. 

### 3.3. Inflammation-Associated miRNAs Are Upregulated in Jejunum of GS Macaques and Have Predicted Binding Sites on Bacterial 16S Ribosomal RNA

**Figure 6:**
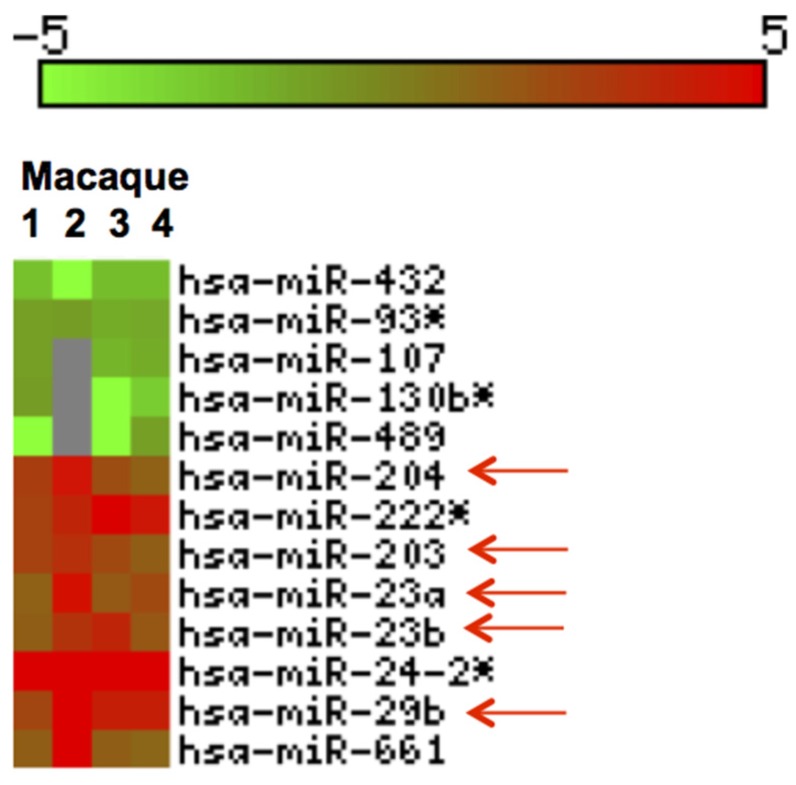
Heat Map of differentially expressed (DE) miRNAs in GS macaques. Values corresponding to jejunum of four GS macaques normalized against average of 5 normal healthy controls are shown. Eight out of 13 DE miRNAs were significantly upregulated (**red**) and the remaining five were downregulated (**green**) (*p* < 0.05). **Red** arrows indicate miRNAs previously reported and linked to inflammatory disorders [55,56,57,58,59,60,61].

We profiled miRNA expression in jejunum of four GS macaques and identified thirteen DE (*p* < 0.05) miRNAs (Figure 6). Out of these, 8 were upregulated and 5 downregulated (Figure 6). 

**Figure 7:**
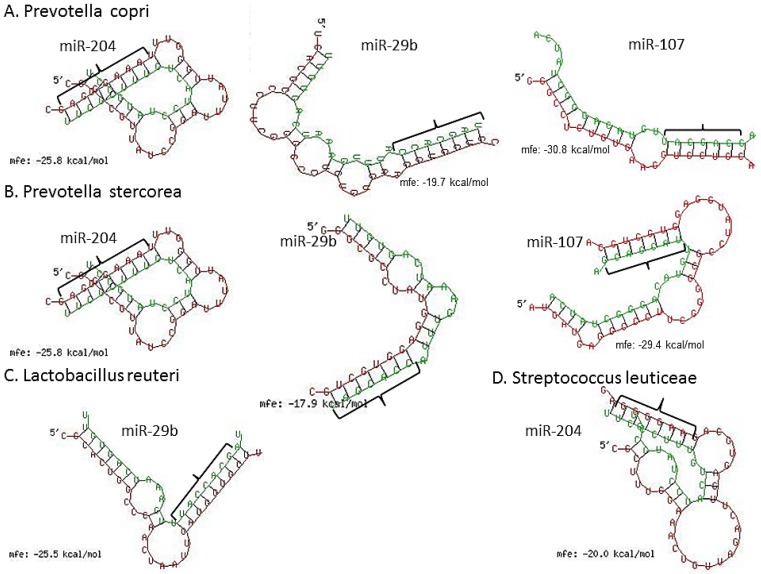
miRNA (**green**) vs. 16S ribosomal RNA (violet) (rRNA) pairing using RNAhybrid algorithm. Note the perfect Watson and Crick base pairing in miRNA 5′ seed nucleotides (nts) 2–7 (bracket) and extra homology in 3′ region of miR-204, miR-29b and miR-107 with 16S rRNA of *P. copri* (**A**); and *P. stercorea* (**B**); In addition, notice the significantly good homology between miR-204 and miR-29b seed nts and 16s rRNA of *L. reuteri* (**C**); and *S. leuticeae* (**D**), respectively.

We next scanned the bacterial 16S rRNA sequence of four bacterial species, namely, *Lactobacillus reuteri* (accession number (No.) NR_025911), *Prevotella copri* (accession No. AB244773), *P. stercorea* (accession No. NR_041364), *Streptococcus luteciae* (accession No. AJ297218) that were found to be overrepresented in feces of GS macaques (Figure 3) for potential binding sites for these DE miRNAs using the RNAhybrid algorithm [62]. As observed and reported previously by Liu et al. [42], using the RNAhybrid algorithm, we identified binding sites for miR-204, miR-29b and miR-107 on the 16S rRNA sequence of *P. copri* and *P. stercorea* that showed perfect Watson and Crick base pairing in the miRNA seed region (nucleotide positions 2 to 7 on 5’ end) with very low minimum free energy (MFE) (Figure 7). Additionally, miR-29b and miR-204 were also found to have binding sites on the 16S rRNA sequence of *L. reuteri* and *S. leuticeae*, respectively. 

### 3.4. Claudin-1, an Epithelial Tight Junction Protein and A Validated Target of miR-29b Is Significantly Downregulated in Jejunum of GS Macaques

MiRNAs and RNA binding proteins are known to regulate TJ protein expression [63,64]. Recent studies in IBD and IBS have demonstrated miR-122 and miR-29b to directly target and downregulate the expression of occludin [65] and claudin-1 [55] expression, respectively. Using the TargetScan 7.1 [66] and RNAhybrid [62] algorithm, we identified a *perfect* Watson-Crick *match* to the *seed nucleotides* 2–7 of three upregulated miRNAs, namely, miR-203, miR-204 and miR-29b on the 3′ UTR of claudin-1 mRNA (Supplementary Materials Figure S3) that are highly conserved across multiple mammalian species that includes chimpanzees and rhesus macaques [66]. These in silico findings strengthen the possibility of direct post-transcriptional silencing of claudin-1 by three different miRNAs. Since miR-29b has already been validated to directly target claudin-1 expression [55], we next investigated claudin-1 protein expression in the jejunum of GS macaques.

**Figure 8:**
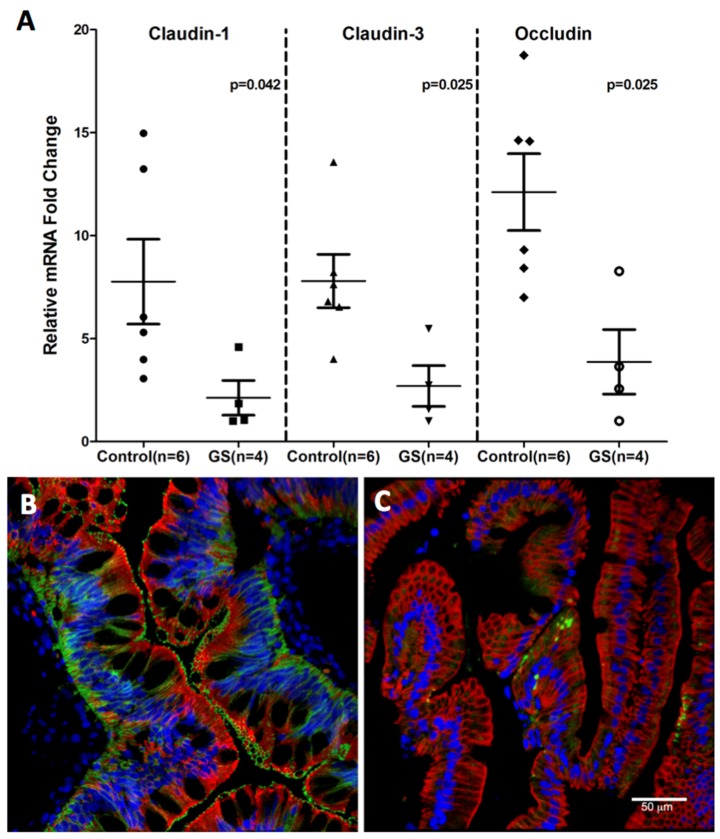
Tight junction protein expression in jejunum of GS macaques. mRNA expression of Claudin-1,-3 and Occludin is significantly decreased (*p* < 0.05) in jejunum of GS (*n* = 4) relative to normal healthy control macaques (*n* = 6) (**A**); Data was normalized to a combination of three endogenous controls (Beta-Actin, 18S rRNA, GAPDH) and analyzed using non-parametric Wilcoxon’s rank sum test for independent samples. The error bars represent standard error of mean fold change within each group. While Claudin-1 (**green**) protein is well expressed in jejunum epithelial cells (**red**) from normal healthy macaque (**B**); its signal is very low or absent in jejunum from GS animal (**C**). Both B and C panels are triple labels with claudin-1 in **green**, cytokeratin in **red** and nuclear labeling with Topro3 in **blue**.

Consistent with miR-203, miR-204 and miR-29b upregulation, downregulation of claudin-1, a TJ protein that regulates intestinal epithelial permeability was observed (Figure 8). The miRNA expression of three TJ proteins, namely, claudin-1, -3 and occludin was significantly diminished in GS relative to normal healthy control macaques (Figure 8A). The decreased RNA expression was corroborated by the confocal microscopy of claudin-1 protein expression (Figure 8B,C) directly in the jejunal villous enterocytes of GS macaque compared to the healthy control. These findings suggest that dysregulated miRNA expression in response to chronic inflammation could enhance epithelial permeability by downregulating TJ protein which in turn would facilitate systemic translocation of dysbiotic bacteria in GS macaques. 

In summary, a non-significant increase in alpha-diversity was observed in GS macaques while on GFD (Supplementary Materials Figure S2A,B), raising the proposition that with further progression of time and continued feeding of GFD, gut microbiomes of GS macaques might revert towards normal, healthy controls. Remarkably, the PERMANOVA results for GFD and RGB time points confirmed that gut microbiome composition (beta-diversity) was changing in GS macaques with progression of time: It was determined that beta-diversity values differed significantly between the time points when GS macaques were switched from gluten-containing to gluten-free (*p* = 0.011) or gluten-reduced (*p* < 0.05) diets. In contrast, alpha-diversity metrics attributed to samples associated with GFD, BOMI and RGB diets did not change significantly during the short-term (1–2.5 months) periods of experimental feeding although their average values were increasing (Supplementary Materials Figure S2C,D). miRNA data demonstrated significant dysregulation in the intestines of GS macaques (Figure 6 and Figure 7) and as previously demonstrated [42], allude to the possibility that dysregulated miRNAs could potentially regulate the intestinal microbiome in GS macaques via post transcriptional gene regulation [42].

## 4. Discussion 

Non-human primates are being used in translational research involving infectious, immune-mediated, metabolic and other disorders where the scientific objectives cannot be fully accomplished by the use of other animal models [43,67,68,69]. The gut microbiomes of two biologically distinct (GS and healthy) groups of captive rhesus macaques were for the first time compared. A recent work by Yasuda and colleagues demonstrated that rhesus stool microbiome is a suitable proxy for both large and small intestine microbiomes [70]. In the present study, representative stool samples were characterized by amplifying V4 region of the 16S rRNA gene [19,32,34,35,37]. It was hypothesized that disease progression in GS macaques is associated with a loss of gut microbial diversity which can potentially lead to increased epithelial permeability thereby exacerbating intestinal inflammation [25,26,27,28,29]. Our findings clearly demonstrate that microbiomes in GS and healthy macaques differ significantly while on long-term (≥one year) conventional, gluten-containing diet, i.e., GD. Since the gut microbiomes of GS macaques have not been studied before, findings reported here are novel and provide directions for potential future studies. The GS macaques can be used in preclinical studies to evaluate if novel dietary or other therapeutic interventions can reverse gut dysbiosis. In studies with unrelated, chronic bacterial colitis-affected macaques, an overgrowth of *Pasteurellaceae* and *Enterobacteriaceae*, as well as decreased microbial diversity was observed. Taken together, our findings also corroborate that gluten sensitivity can contribute to chronic bacterial enterocolitis e.g., one of the major health concerns of polyfactorial origin in captive macaques [24,44,71,72]. 

As noted by McKenna and colleagues [19], a distinctive feature of the macaque gut microbiome is the abundance of intestinal Spirochetes from the *Treponema* lineage. In agreement with those observations, in the present study, intestinal Spirochetes were abundant in healthy controls, while GS macaques had lower loads of these bacteria. This finding suggests that a thriving population of intestinal Spirochetes is indeed an indicator of robust health in macaques. It is also consistent with the findings of Zeller and Takeuchi [73], who pointed out the presence of intestinal Spirochetes in healthy macaques. Our group previously reported that intestinal Spirochetes, despite their high prevalence, were not among intestinal bacteria linked with chronic enterocolitis [44]. 

One of the key similarities between human and rhesus gut microbiomes is that *Firmicutes* and *Bacteroidetes* are the two prominent phyla. It was established that the ratio between these two can be in humans affected by “western” and “low-calorie/high-fiber” types of diets [74,75]. Consistent with these findings, *Firmicutes* followed by *Bacteroidetes*, were amongst the most abundant phyla represented in our study macaques. Nonetheless, several differences in composition were observed between GS and healthy control macaques. While GS macaques exhibited dysbiosis, several groups of intestinal bacteria were differentially abundant when compared with healthy controls. The over-abundant groups included two major families belonging to the phylum *Firmicutes*, i.e., *Streptococcaceae* and *Lactobacillaceae*. Previously, it was reported by Caminero et al., that both *Lactobacillaceae* and *Streptococcaceae* play an important role in metabolism of gluten [39]. While it is obvious that the presence of *Streptococcaceae* represents potential to contain pathogenic strains, the biological significance underlying the increased presence of *Lactobacillaceae* in GS macaques is less certain. Clearly, *Lactobacillus* spp. have the capacity to degrade gluten resulting in decreased immunotoxicity of its major immunogens such as the 33-mer of alpha-gliadin [76]. At the same time, however, the full pathogenic potential of dysbiotic bacterial taxa including *Lactobacillaceae*, *Streptococcaceae* and others in GS individuals still needs to be elucidated. Interestingly, and in concordance with our study, Ardeshir and colleagues (2014) independently reported that chronic intestinal enterocolitis is in rhesus macaques associated with an over-abundance of intestinal *Lactobacillaceae* [24], suggesting that not all of the *Lactobacilli* spp. act as a health-promoting probiotics. According to their study, *Lactobacillaceae* overgrowth can be reduced in macaques by inulin treatment [24]. Less abundant taxa in GS macaques were mostly represented by *Coriobacteriaceae* that belong to phylum *Actinobacteria*. *Actinobacteria* were recognized as the producers of host-beneficial metabolites with antibacterial, antifungal, immunomodulatory and other functions [66,77]. Reduced abundance of *Bacteroidetes* has been previously reported in human celiac infants [33]. Similar studies that utilized different technologies, and focused on different types of (biopsy) samples, have not always produced consistent results [32,34,35,37]. In our study, a few of the bacterial taxa belonging to *Bacteroidetes* were less abundant in GS macaques while others, namely *Prevotella* sp., were overabundant compared to healthy controls. One group of patients where GS occurs with higher frequency and in parallel with neurodevelopmental disorders, are the patients with Autism Spectrum Disorder [78,79]. It has been reported that Autism and Parkinson’s disease patients lack beneficial gut microflora [80,81]. In this context, we previously reported that up-regulation of the Autism Spectrum Disorder-associated gene CADPS2, and other neurodevelopmental disorder-related genes (BACE2 and DSCR5) were detected in GS macaques [82]. Despite that these associations and links are still largely under-explored, they offer clues for potential future studies. 

While microbial dysbiosis is a hallmark of chronic inflammatory diseases of the gastrointestinal tract, the potential mechanisms underlying these alterations remain unknown. A recent study demonstrated that fecal miRNAs secreted by intestinal epithelial cells could enter luminal bacterial cells and regulate their growth via post-transcriptional gene regulation [42]; suggesting a critical mechanism by which the host could not only shape but also potentially dysregulate its intestinal microbiome. Additionally, miRNAs have also been demonstrated to regulate the intestinal epithelial barrier in inflammatory bowel disease (IBD) and irritable bowel syndrome (IBS) via post-transcriptional regulation of TJ proteins [54,83]. Notable miRNAs associated with inflammation in our study included miR-203 and miR-29b that have previously been reported to be upregulated in IBD and IBS [54,55,56]. More importantly, the inverse relationship between miR-203, -204 and -29b expression and their predicted/validated claudin-1 target protein expression, suggests an important post-transcriptional mechanism regulating the intestinal epithelial barrier that could promote translocation of dysbiotic intestinal bacteria leading to adverse systemic inflammation/immune dysregulation in GS macaques and celiac disease patients. Similarly, dysregulation of miR-204 and miR-23a/b has been reported in various other inflammatory conditions [58,59,60,61].

Although we identified several DE miRNAs previously associated with various chronic inflammatory diseases to have binding sites on the 16S rRNA sequence of *Lactobacillus*, *Prevotella* and *Streptococcus* species in GS macaques with gut dysbiosis, further studies are needed to corroborate presence of these miRNAs in feces of GS individuals and to correlate their expression levels with changes in the bacterial flora. Data from such analyses will pave the way for in vitro mechanistic/growth kinetic studies [42] to elucidate the novel concept of whether dietary gluten-induced dysbiosis involves selective modulation of the GI microbiota via luminal shedding of intestinal epithelial miRNAs.

## 5. Conclusions

This is the first report illustrating the reduction of gut microbial diversity following the consumption of dietary gluten in GS macaques. Although administration of GFD to GS macaques was expected to restore composition of dysbiotic gut microbiomes to normal, diversity metrics did not corroborate such expectation. These findings are consistent with studies of celiac patients whose gut microbiome composition was not restored even after “long-term” treatment with GFD [84]. Notwithstanding, we believe that further extension of GFD feeding regimen and/or inclusion of additional treatments such as anti-inflammatory compounds would result in more complete restoration of intestinal microbiota in GS subjects. Thus, the present study has set the stage for future experiments, in which the effects of novel treatment strategies will be assessed. These approaches will include oral probiotics, microbiome restitution, gluten-modified diets, recombinant glutenases and anti-inflammatory drugs. The gut microbiome and miRNA metrics are expected to provide useful evaluative tools in these studies. 

## Supplementary Materials

The following are available online at http://www.mdpi.com/2072-6643/8/11/684/s1, Figure S1: Hierarchical clustering by GS status. Microbiomes were clustered by the Ward Method and Bray-Curtis Distance. The two clusters (control healthy = blue and GS = green) of macaques, all fed GD diet, are differentiated by GS status, except for KM76. KM76 macaque had a lower proportion of Strepococcaceae (6.8%), similar to healthy controls, Figure S2: Alpha-diversity estimates (Observed and Shannon) in GS macaques while on GFD (A,B); as well as comparisons between BOMI, GFD and RGB diets (C,D), Figure S3: miRNA (green) vs. claudin-1 mRNA 3′ UTR (red) pairing using RNAhybrid algorithm. Note the perfect Watson and Crick base pairing in miRNA 5′ seed nucleotides (nts) 2–7 (bracket) and extra homology in 3′ region of miR-203, miR-204 and miR-29b with claudin-1 mRNA 3′ UTR, Table S1: Rhesus macaque stool sample descriptions, Table S2: Primer sequences used for real time SYBR Green One-step qRT-PCR. Table S3. Kruskal-Wallis rank sum test—alpha-diversity metrics, Table S4: Kruskal-Wallis rank sum test—8 most abundant families, Table S5: Selected time points of GFD: D14–D70.
