# Supplementary material for: Dietary Gluten-Induced Gut Dysbiosis Is Accompanied by Selective Upregulation of microRNAs with Intestinal Tight Junction and Bacteria-Binding Motifs in Rhesus Macaque Model of Celiac Disease
Source: Nutrients. 2016 Oct 28;8(11):684. doi: 10.3390/nu8110684 (PMC5133072; doi:10.3390/nu8110684)
Supplement: Supplementary file 1 [file nutrients-08-00684-s001.docx]

Supplementary Materials: Dietary Gluten-Induced Gut Dysbiosis Is Accompanied by Selective Upregulation of microRNAs with Intestinal Tight Junction and Bacteria-Binding Motifs in Rhesus Macaque Model of Celiac Disease

Mahesh Mohan, Cheryl-Emiliane T. Chow, Caitlin N. Ryan, Luisa S. Chan, Jason Dufour,
Pyone P. Aye, James Blanchard, Charles P. Moehs and Karol Sestak

**Table S1.** Rhesus macaque stool sample descriptions.


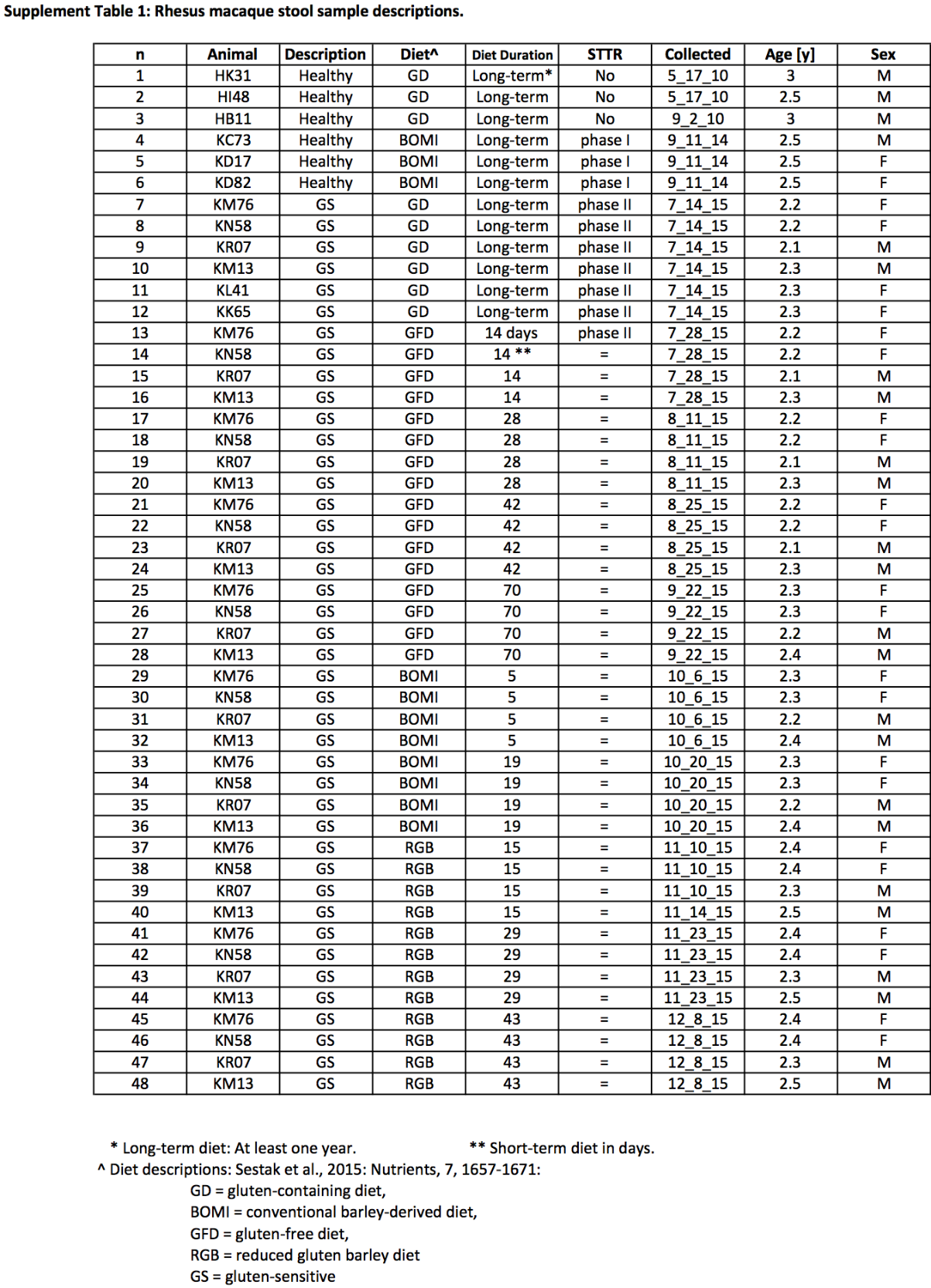


**Table S2.** Primer sequences used for real time SYBR Green One-step qRT-PCR.


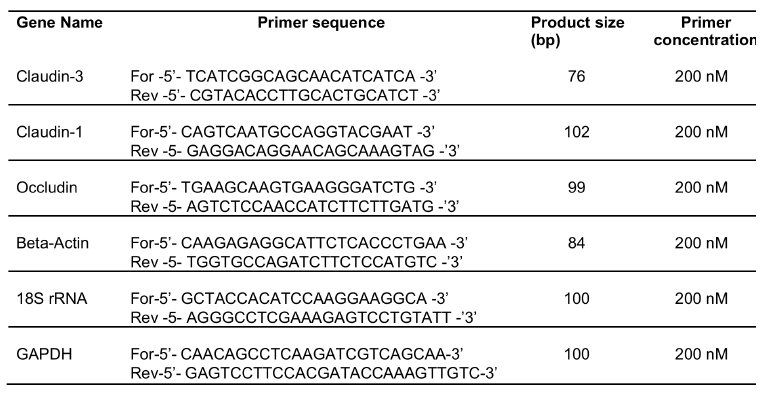


**Table S3.** Kruskal-Wallis rank sum test—alpha-diversity metrics.


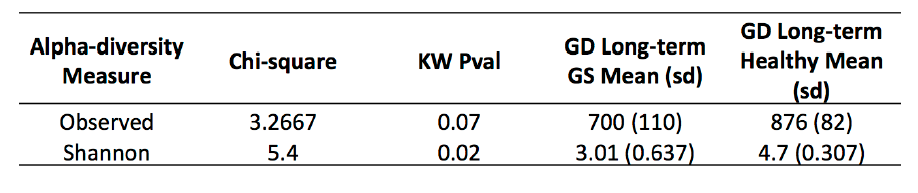


**Table S4.** Kruskal-Wallis rank sum test—8 most abundant families.


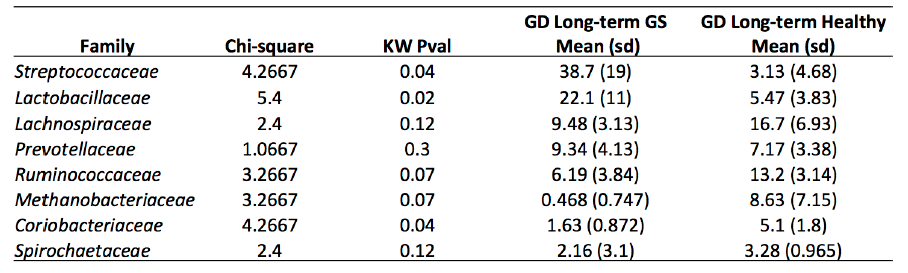


**Table S5.** Selected time points of GFD: D14–D70.


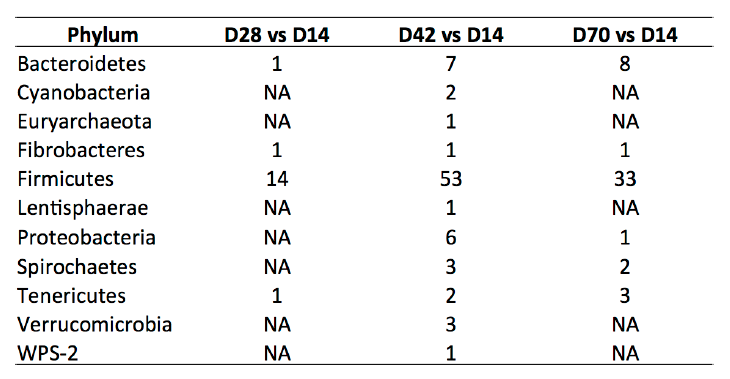


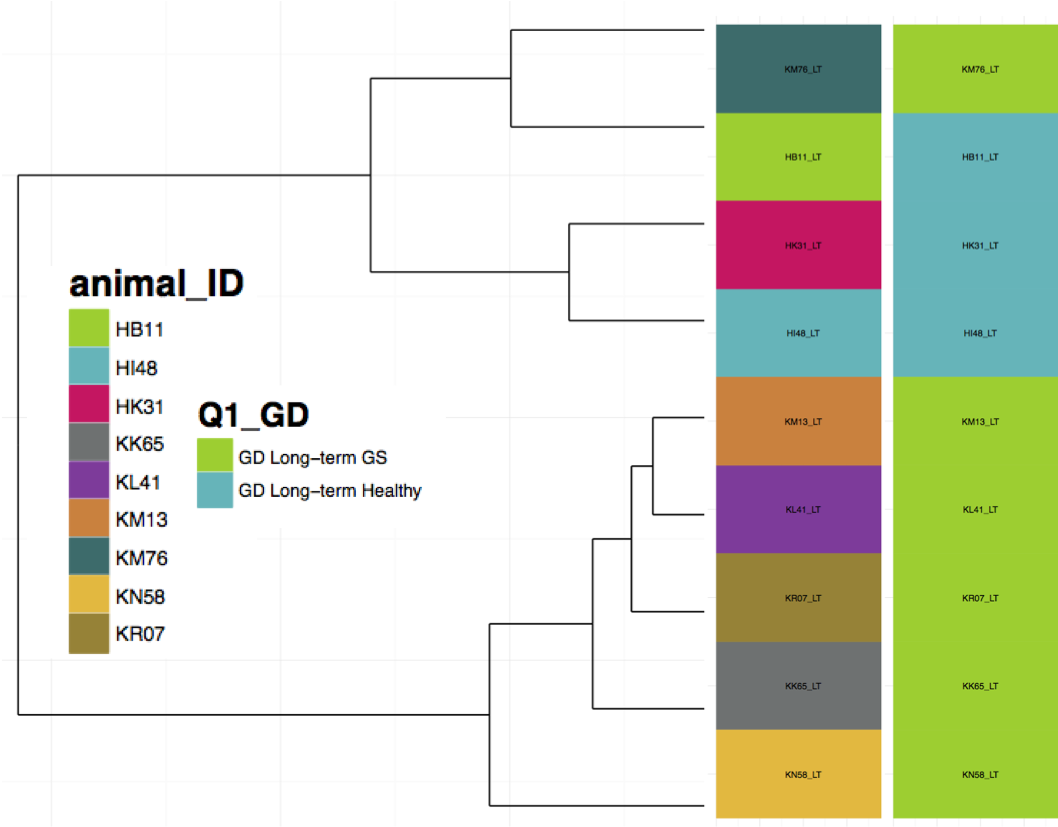


**Figure S1.** Hierarchical clustering by GS status. Microbiomes were clustered by the Ward Method and Bray-Curtis Distance. The two clusters (control healthy = **blue** and GS = **green**) of macaques, all fed GD diet, are differentiated by GS status, except for KM76. KM76 macaque had a lower proportion of *Strepococcaceae* (6.8%), similar to healthy controls.


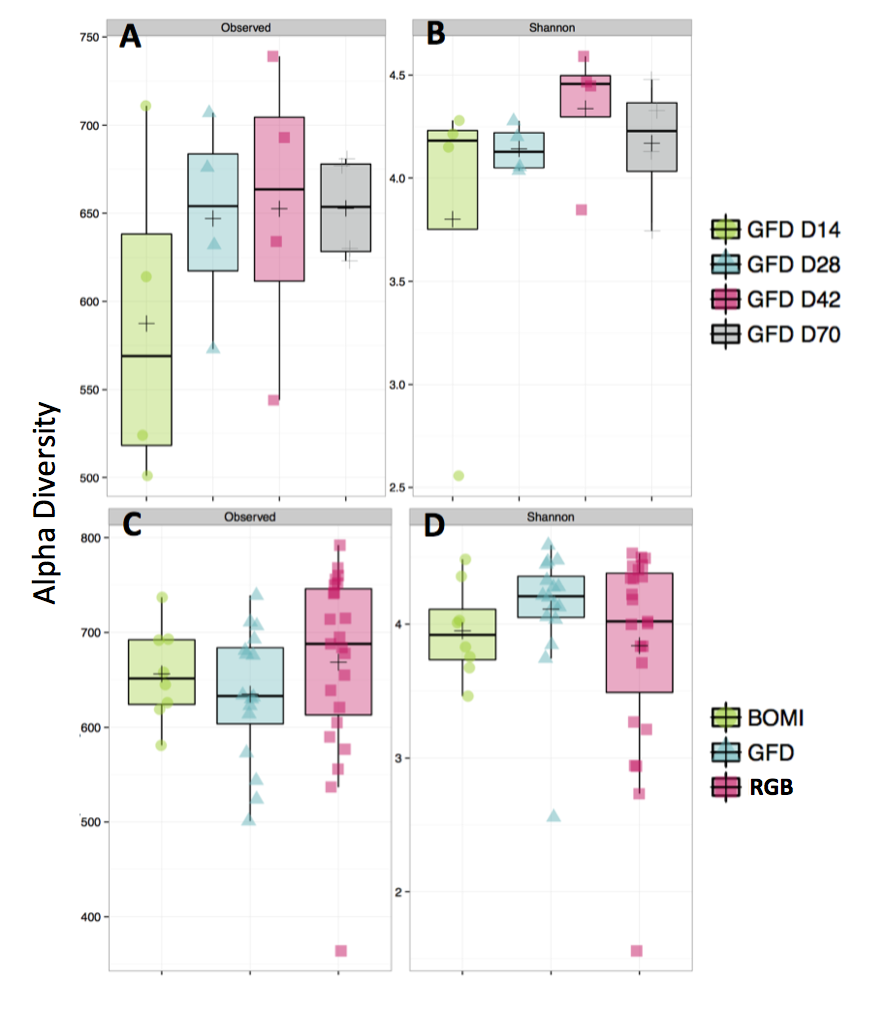


**Figure S2.** Alpha-diversity estimates (Observed and Shannon) in GS macaques while on GFD (**A**,**B**); as well as comparisons between BOMI, GFD and RGB diets (**C**,**D**).


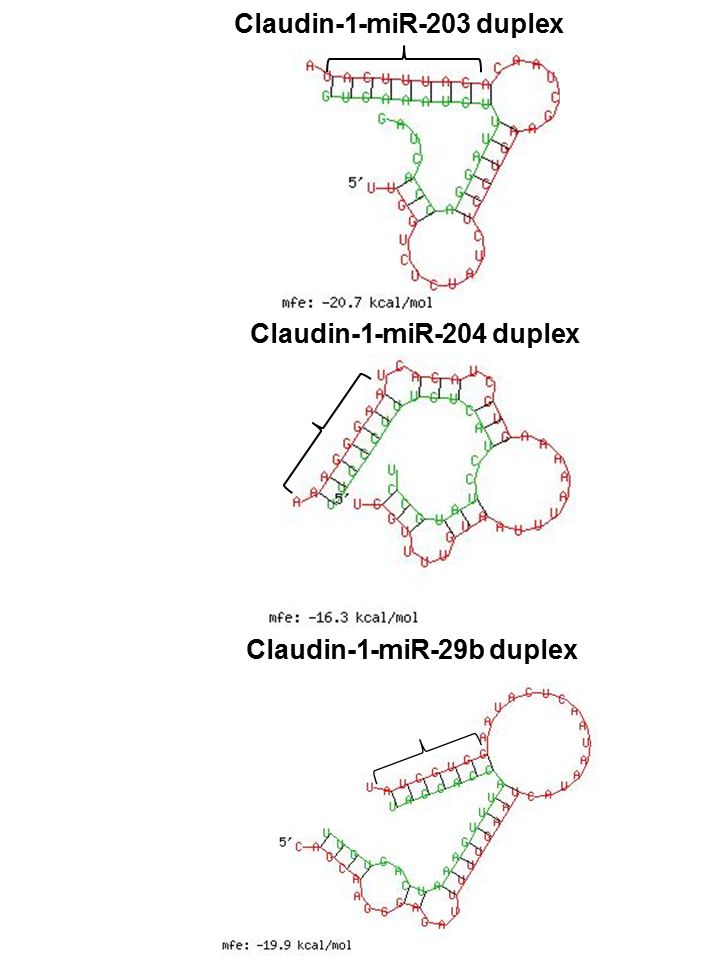


**Figure S3.** miRNA (**green**) vs. claudin-1 mRNA 3′ UTR (**red**) pairing using RNAhybrid algorithm. Note the perfect Watson and Crick base pairing in miRNA 5′ seed nucleotides (nts) 2–7 (bracket) and extra homology in 3′ region of miR-203, miR-204 and miR-29b with claudin-1 mRNA 3′ UTR.
